# Gastrointestinal Carriage of Antimicrobial Resistance in School-Aged Children in Three Municipalities of Timor-Leste

**DOI:** 10.3390/antibiotics11091262

**Published:** 2022-09-16

**Authors:** Tessa Oakley, Brandon Le, Virginia da Conceicao, Ian Marr, Carolina Maia, Messias Soares, Joana Correia Belo, Nevio Sarmento, Endang da Silva, Salvador Amaral, Susana Vaz Nery, Sarah Lynar, Joshua R. Francis, Jennifer Yan

**Affiliations:** 1 Global and Tropical Health Division, Menzies School of Health Research, Charles Darwin University, Dili, Timor-Leste; 2 The Kirby Institute, University of New South Wales, Sydney 2052, Australia; 3 The Canberra Hospital, Canberra 2605, Australia; 4 National Health Laboratory, Dili, Timor-Leste; 5 Royal Darwin Hospital, Darwin 0810, Australia

**Keywords:** AMR, colonisation, multidrug-resistant, *E. coli*, Enterobacterales, ESBL, third-generation cephalosporins

## Abstract

Invasive bacterial infections are a leading cause of death in children, primarily in low- and middle-income countries (LMIC). Links between carriage of antimicrobial-resistant organisms and more resistant infections have been established; however, little has been reported regarding community carriage of antibiotic-resistant organisms such as extended-spectrum β-lactamase (ESBL)-producing Enterobacterales in LMIC. The aim of this study was to determine colonic carriage of ESBL-producing fluoroquinolone- and aminoglycoside-resistant Enterobacterales in healthy children in three municipalities of Timor-Leste. In November 2020, 621 stool samples were collected from school-aged children and underwent screening for the presence of Enterobacterales species and antimicrobial resistance (AMR). Ciprofloxacin-resistant Gram-negative organisms were cultured from 16.5% (95% CI 6.2–26.9), and gentamicin resistance was identified in 6.8% (95% CI 2.8–10.7). Compared to the prevalence of ciprofloxacin resistance in Dili (36.1%), there was significantly lower prevalence in the rural municipalities of Ermera (12.9%; AOR 0.38, 95% CI 0.24–0.60, *p* < 0.001) and Manufahi (4.5%; AOR 0.07, 95% CI 0.01–0.51, *p* = 0.009). The overall cluster-adjusted prevalence of ESBL-producing bacteria was 8.3%, with no significant differences between municipalities. This study demonstrates high rates of carriage of AMR among school-aged children in Timor-Leste, with higher rates observed in Dili compared to rural municipalities. Empiric antibiotic guidelines should include recommendations for treating community-acquired infections that account for the possibility of antimicrobial resistance.

## 1. Introduction

Timor-Leste is a Southeast Asian nation situated in the Malay Archipelago with a population of 1.3 million people [1]. The country consists of 13 municipalities, with mountainous terrain. Invasive bacterial infections are a leading cause of death in children, especially in low- and middle-income countries (LMIC), including Timor-Leste [2,3]. Mortality due to infections with bacterial antimicrobial resistance (AMR) is increasing and is recognised as a major global health issue, with an estimated 4.95 million deaths associated with bacterial AMR in 2019 [4,5]. AMR in Gram-negative organisms is associated with an increasing burden of disease; however, limited AMR surveillance systems and a lack of local data frequently make it challenging to estimate drug resistance rates in LMIC [6,7,8].

Broad-spectrum antibiotics such as third-generation cephalosporins (3GC) are commonly prescribed to treat infections caused by Enterobacterales organisms [9]. Increasing rates of extended-spectrum β-lactamase (ESBL) producers in Enterobacterales have been described over the past two decades, leading to a rise in treatment failures with 3GC [10]. ESBL-producing organisms also commonly carry resistance genes to other classes of antibiotics, including fluoroquinolones and aminoglycosides, posing challenges in determining appropriate antibiotic therapy [11,12]. Independent of the site of infection, the digestive tract appears to be the main reservoir for ESBL-producing organisms [13]. While a better understanding of the impact of colonic carriage of resistance is needed, some research has highlighted carriage as a predisposition towards more resistant infections and a poorer prognosis with sepsis episodes [14,15,16].

AMR surveillance in LMIC is primarily conducted passively, with data obtained from clinical samples [17,18]. However, the use of clinical samples alone for AMR surveillance may misrepresent AMR community carriage rates, due to the underutilisation of diagnostic microbiology services in many LMICs and the fact that bacterial culture is often not performed until after the initial treatment has failed [17,19]. Community transmission is a known risk factor for AMR spread in Southeast Asia [20]. In areas where diagnostic microbiology capacity is limited, active AMR carriage surveillance, such as stool screening for Gram-negative organisms, can be utilised as an alternative or in addition to passive surveillance [21,22,23].

Only one study has previously been performed documenting AMR rates in Timor-Leste which analysed 211 urine and skin isolates from a Dili hospital and found that, of the Enterobacterales species isolated, 35% were resistant to ceftriaxone with an ESBL-producing phenotype [24]. There have been significant capacity-building efforts in diagnostic microbiology at the National Health Laboratory (NHL) in the capital city of Dili; however, knowledge of Gram-negative resistance in other municipalities is limited. Previous studies have shown that school children can be colonised with the Enterobacterales organism *E. coli*, resistant to broad-spectrum cephalosporins and ciprofloxacin [25,26]. However, no studies have been performed on AMR carriage in school-aged children in Timor-Leste.

In the context of an impact assessment of the Timor-Leste Ministry of Health’s 2019 mass drug administration (MDA) program for lymphatic filariasis (LF) on STH and scabies, we collected additional stool samples to assess AMR carriage rates in school-aged children in three municipalities of Timor-Leste. This study aims to improve our understanding of the epidemiology and geographical distribution of Gram-negative resistance carriage outside the capital city of Dili and utilise this information to better inform empiric antibiotic guidelines.

## 2. Materials and Methods

**Figure 1:**
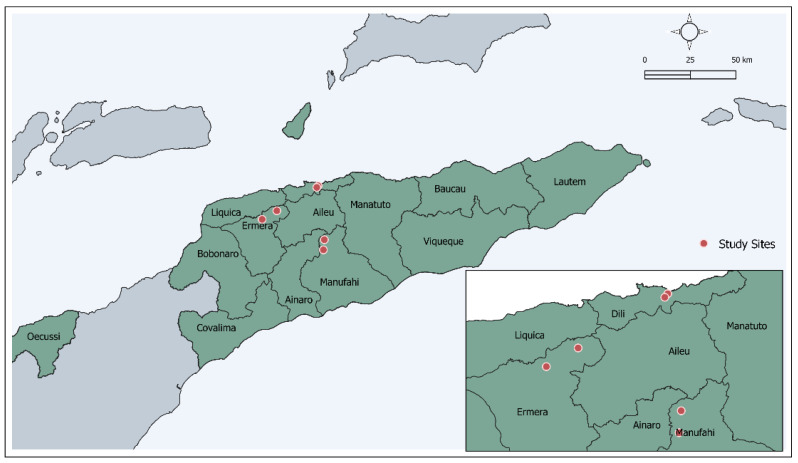
Map of sampling locations in Timor-Leste. The figure shows locations of the six primary schools, indicated by red pins, in the three municipalities included in the study.

A cross-sectional survey was conducted in six schools across three municipalities of Timor-Leste, one of which was urban (Dili) and two rural (Ermera, Manufahi) (Figure 1). The survey was performed in the context of an impact assessment MDA conducted by the Timor-Leste Ministry of Health in 2019 for lymphatic filariasis (LF) control [27]. As part of this process, a baseline assessment of the impact of the MDA on scabies and soil-transmitted helminths was conducted, followed by an 18-month follow-up assessment in November 2020.

Two preparatory visits were completed prior to the study. The first involved requesting approval from the school principal for the study to take place in their school. The second was to inform the teachers of the study and to ask them to inform parents to attend school on an agreed date for our team to present information about the study. On day 1, the local project manager provided a presentation to parents about the study, included the opportunity to ask questions, and then sought parental written consent for stool collection. The team visited each classroom, registering students whose parents provided consent to stool collection. The project manager gave a presentation to students on how to provide a stool sample and then distributed stool collection kits containing a plastic specimen collection container, gloves, a plastic spoon, and a study information sheet. Students whose parents provided written consent were asked to return their samples to the team the following day. All students whose parents provided written consent for stool screening were eligible to participate in the present study. One aliquot of stool (3 g each) per participant was collected for AMR screening, fixed in Copan FecalSwab (Copan Diagnostics, Murrieta, CA) and stored at 2–8 °C before transport to Dili.

**Figure 2:**
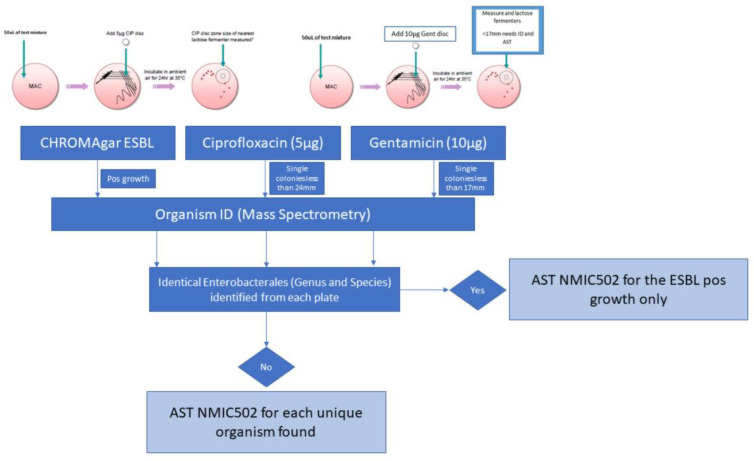
Job aid used for stool sample processing.

Samples were streaked using a sterile 10 µL loop to selective ESBL agar (CHROMagar, Paris, France). Media were provided as dehydrated powders and prepared in-house according to the manufacturer’s instructions. In-house ESBL verification occurred against reference strains *E. coli* ATCC 35218 (beta-lactamase-producing strain) and *E. coli* ATCC 25922 (non-beta-lactamase-producing strain) (KWIK-STIK, Microbiologics, St Cloud, MN, USA). Concurrently, 50 µL of vortexed stool solution was pipetted onto two MacConkey agar plates and streaked. Screening for ciprofloxacin resistance was performed using a 5 µg ciprofloxacin disc, and screening for isolated gentamicin resistance was performed using a 10 µg gentamicin disc (BD BBL™ Sensi-Disc™). All plates were incubated overnight at 37 °C (Figure 2).

All isolates with growth on CHROMAgar ESBL or within ciprofloxacin and gentamicin screening zones were identified with matrix-assisted laser desorption/ionisation time-of-flight mass spectrometry (MALDI-ToF MS) (Bruker Daltonik, Bremen, Germany). Those confirmed as Enterobacterales underwent AST analysis utilising BD Phoenix NMIC-502 panels (Becton Dickinson, Berks, UK). European Committee on Antimicrobial Susceptibility Testing (EUCAST) minimum inhibitory concentration breakpoints were used to classify isolates as either susceptible or resistant [28]. Identical isolates found by MS from separate plates (ESBL, fluoroquinolone, or aminoglycoside screening plates) were processed only once via BD Phoenix NMIC-502 AST. Where two or more unique isolates within the same stool sample were identified, the isolate found with higher co-resistance was included for that individual.

Mixed-effects generalised linear models were used to calculate the prevalence of faecal carriage of ESBL-producing Enterobacterales and resistance to antimicrobial agents among children, adjusting for school-level clustering via random effect terms. These models were also used to compare differences in odds of faecal carriage of ESBL and antimicrobial resistance between municipalities. Adjusted odds ratios (AORs) were adjusted for school-level clustering, sex, and age. Cases with missing sex or age data were excluded from the analysis. Statistical analysis was completed using Stata version 14.2 (StataCorp, College Station, TX, USA).

## 3. Results

A total of 621 children provided a single stool sample. Half of the participants were from semiurban Dili (50.4%), compared to rural sites (Ermera 42.2%, Manufahi 7.4%). Where data were available, 54.7% were boys, and 45.3% were girls. The median age was 10 years (IQR 9–12). There were 242 bacterial isolates identified as screening test positive from the 621 children. Most were *E. coli* (92.6%), with smaller numbers of *Pseudomonas* sp. (4.5%), *Acinetobacter* sp. (1.7%), and one case each of Klebsiella pneumoniae (0.4%), *Psychrobacter* sp. (0.4%), and Raoultella ornithinolytica (0.4%). Ciprofloxacin resistance was identified in 18.5% of participants and gentamicin resistance in 7.4%. The unadjusted prevalence of ESBL-producing bacteria was 10.1% (Table 1). Of the 136 isolates from stool samples that were screening test positive for gentamicin resistance, 41 exhibited resistance when tested by conventional AST (30.1%). The use of the ciprofloxacin disc screening method resulted in 122 isolates selected for further testing, of which 115 were found to be ciprofloxacin-resistant via conventional AST (94.3%).

Table 2 summarises the cluster-adjusted prevalence of faecal carriage of ESBL-producing bacteria and resistance to antimicrobial agents and differences between municipalities. The overall cluster-adjusted prevalence of resistance to ciprofloxacin was 16.5% (95% CI 6.1–26.9), and resistance to gentamicin was 6.8% (95% CI 2.8–10.7). Compared to the prevalence of ciprofloxacin resistance in Dili (36.1%), there was a significantly lower prevalence in Ermera (12.9%; AOR 0.38, 95% CI 0.24–0.60, *p* < 0.001) and Manufahi (4.5%; AOR 0.07, 95% CI 0.01–0.51, *p* = 0.009). Similarly, relative to the prevalence of gentamicin resistance in Dili (11.8%), there was a significantly lower prevalence in Ermera (5.2%; AOR 0.40, 95% CI 0.20–0.81, *p* = 0.011). The overall cluster-adjusted prevalence of ESBL-producing bacteria was 8.3% (95% CI 1.6–15.1). There was no statistically significant difference in ESBL prevalence between municipalities (Dili 13.4% vs. Ermera 8.4%; AOR 0.80, 95% CI 0.47–1.38, *p* = 0.429); however, no cases with ESBL carriage were identified in 46 participants from Manufahi.

## 4. Discussion

In this study, we described the community gastrointestinal carriage of AMR in Enterobacterales species in school-aged children in Timor-Leste. Ciprofloxacin resistance was identified in nearly one-fifth of participants; however, carriage of gentamicin resistance (7.4%) and ESBL-producing Enterobacterales (10.1%) was less common. The association of ESBL production with fluoroquinolone resistance is of concern, with nearly half of the ESBL-producing isolates in this study also exhibiting resistance to ciprofloxacin, while approximately 10% of ESBL-producing isolates also demonstrated gentamicin resistance.

The results of this study align with a previous report on global ESBL carriage at 14% prevalence amongst healthy individuals [29]. High rates of ESBL-carrying Enterobacterales were observed in a previous clinical study in Timor-Leste, with an analysis of Gram-negative isolates from clinical urine samples demonstrating a 35% phenotypic ESBL production [24]. Ciprofloxacin and gentamicin resistance rates were higher in Dili than in other municipalities, possibly due to increased antibiotic exposure as a result of more accessible over-the-counter antibiotics. However, data on antimicrobial consumption in health facilities in Timor-Leste do not suggest higher use in Dili [30]. Higher rates of Gram-negative resistance may also be influenced by greater transmission pressure related to higher population density [31].

Although the use of a ciprofloxacin disc for the screening of fluoroquinolone resistance in faecal samples has been previously described, this was the first known attempt to screen for aminoglycoside resistance using a gentamicin disc [32,33]. Gentamicin resistance in Enterobacterales has implications for empiric sepsis treatment guidelines. There are no current validated methods for determining aminoglycoside resistance in rectal swabs. Potential overgrowth by *Enterococci* sp. meant that MAC agar with a gentamicin disc was chosen as a method for screening out Gram-positive organisms from cultured stool samples. This method lacked specificity for identifying gentamicin resistance, with true gentamicin resistance identified by conventional AST for less than a third of Enterobacterales which tested positive by the disc screening method. Further research should be carried out to determine an effective screening test for aminoglycoside resistance in stool samples.

There are several limitations to this study. First, geographical coverage of the sample collection sites was limited, with only three of the thirteen municipalities of Timor-Leste included in the study. Secondly, the sample population consisted only of school-aged children, and so the accuracy of application of these results to community AMR carriage rates amongst adult populations cannot be assumed. Thirdly, information on antimicrobial exposure amongst the study participants was not obtained, which could impact on AMR carriage and confound results. Further research on AMR carriage in an expanded population including additional age groups and municipalities would be beneficial. Medical documentation relating to antimicrobial treatment amongst study participants would allow for more accurate data; however, collection of this information is difficult, due to limited prescribing records in the country.

Whilst these findings provide useful information to guide public health strategies, ongoing AMR surveillance in both clinical and community samples is recommended to monitor changing trends. An effective diagnostic microbiology service is an essential contribution to AMR surveillance, and recent efforts to strengthen laboratory services in-country have contributed to an increased understanding of clinical AMR rates. Further strengthening of AMR surveillance in the environmental and animal health sectors will contribute to a better understanding of the community AMR burden in Timor-Leste.

## 5. Conclusions

This study identified high rates of carriage of ESBL-producing fluoroquinolone- and aminoglycoside-resistant Enterobacterales in healthy children in Timor-Leste, with some evidence of geographic variation in carriage rates. Understanding AMR carriage rates is important for the consideration of empiric treatment recommendations, especially for community-acquired Gram-negative sepsis. Further similar surveys could be conducted to monitor changes in AMR carriage rates over time in response to antimicrobial stewardship and other public health interventions.

## Figures and Tables

**Table 1:** Unadjusted prevalence of antimicrobial resistance and ESBL by municipality.

	Sample Size N	Ciprofloxacin Resistance *n* (%)	Gentamicin Resistance *n* (%)	ESBL *n* (%)	ESBL with Ciprofloxacin Resistance *n* (%)	ESBL with Gentamicin Resistance *n* (%)	ESBL with Ciprofloxacin and Gentamicin Co-Resistance *n* (%)
Sex *							
Male	273	49 (17.9)	26 (9.5)	32 (11.7)	15 (5.5)	5 (1.8)	1 (0.4)
Female	330	59 (17.9)	16 (4.8)	29 (8.8)	9 (2.7)	1 (0.3)	0
Age group (years) *							
≤10	339	56 (16.5)	26 (7.7)	38 (11.2)	14 (4.1)	5 (1.5)	0
>10	264	52 (19.7)	16 (6.1)	23 (8.7)	10 (3.8)	1 (0.4)	1 (0.4)
Municipality							
Dili	313	83 (26.5)	33 (10.5)	37 (11.8)	18 (5.7)	3 (1.0)	1 (0.3)
Ermera	262	30 (11.4)	12 (4.6)	26 (9.9)	7 (2.7)	3 (1.1)	0
Manufahi	46	2 (4.3)	1 (2.2)	0	0	0	0
Total	621	115 (18.5)	46 (7.4)	63 (10.1)	25 (4.0)	6 (1.0)	1 (0.2)

* Sex and age were not recorded for 18 participants (2.90%).

**Table 2:** Cluster-adjusted prevalence of antimicrobial resistance and ESBL by municipality.

	Sample N	Ciprofloxacin Resistance		Gentamicin Resistance		ESBL	
		% (95% CI)	AOR (95% CI)	% (95% CI)	AOR (95% CI)	% (95% CI)	AOR (95% CI)
Dili	313	36.09 (27.03–45.14)	Ref	11.79 (7.53–16.04)	Ref	13.41 (8.81–18.01)	Ref
Ermera	262	12.93 (8.01–17.84)	0.38 (0.24–0.60) *p* < 0.001	5.20 (0.83–9.57)	0.40 (0.20–0.81) *p* = 0.011	8.39 (0–16.88)	0.80 (0.47–1.38) *p* = 0.429
Manufahi	46	4.54 (0–10.99)	0.07 (0.01–0.51) *p* = 0.009	2.22 (0–6.63)	NA, *n* = 1	0	NA
Total	621	16.52 (6.14–26.91)		6.76 (2.83–10.69)		8.30 (1.56–15.05)	

## Data Availability

Data available on request due to restrictions, e.g., privacy or ethical. The data presented in this study are available on request from the corresponding author.
